# Sleep patterns and potential risk factors for disturbed sleep quality in patients after surgery for infective endocarditis

**DOI:** 10.1186/s13019-022-01828-4

**Published:** 2022-05-17

**Authors:** Xiang-Ming Hu, Wen-Ting Wei, De-Yi Huang, Cai-Di Lin, Fen Lu, Xiao-Ming Li, Huo-Sheng Liao, Zhi-Hong Yu, Xiao-Ping Weng, Shi-Bin Wang, Cai-Lan Hou, Fu-Jun Jia

**Affiliations:** 1 Department of Comprehensive Medicine, Guangdong Provincial People’s Hospital, Guangdong Academy of Medical Sciences, Guangzhou, Guangdong Province China; 2 Guangdong Mental Health Center, Guangdong Provincial People’s Hospital, Guangdong Academy of Medical Sciences, No. 123 Huifu Road, Yuexiu District, Guangzhou, 510080 Guangdong Province China

**Keywords:** Infective endocarditis, Sleep quality, Actigraphy, The Pittsburgh sleep quality index (PSQI), The Epworth sleepiness scale (ESS)

## Abstract

**Background:**

The current study aimed to investigate the sleep quality of patients after valve replacement surgery due to infective endocarditis and identify risk factors for disturbed sleep post hospitalisation.

**Methods:**

Eighty patients were assessed postoperatively using subjective scale measures, the Pittsburgh sleep quality index (PSQI) and the Epworth sleepiness scale, and an objective measure, actigraphy. Scale measures were assessed approximately 2 weeks and 6 months after surgery. Actigraphy monitoring was performed for 2 consecutive weeks during hospitalisation. Logistic regression was used to identify risk factors for disturbed sleep.

**Results:**

The study population (n = 80) had an average age of 42.8 ± 14.2 years, and 67.5% were male. The median sleep efficiency was 85.3% in week 1 and 86.8% in week 2. The frequency of awakenings was significantly higher in week 1 (20.0 times vs. 19.3 times, *p* = 0.017). The scale measures showed significant improvement in sleep by 6 months after surgery compared to that during hospitalisation. Multivariable logistic regression analysis suggested that the possible risk factors for disturbed sleep 6 months after surgery included age (OR = 1.479, 95%CI 1.140–1.920) and a few parameters of early postoperative disturbed sleep quality (PSQI: OR = 2.921, 95%CI 1.431–5.963; sleep efficiency: OR = 0.402, 95%CI 0.206–0.783; and average duration of awakenings: OR = 0.006, 95%CI 0.000–0.827).

**Conclusions:**

Disturbed sleep quality was witnessed in postoperative patients during hospitalisation and up to 6 months after surgery. Over time, the patients’ sleep quality improved significantly. Age and a few early postoperative sleep quality variables were risk factors for disturbed sleep 6 months after surgery.

**Supplementary Information:**

The online version contains supplementary material available at 10.1186/s13019-022-01828-4.

## Background

Research of the impact of cardiac surgery on patients’ postoperative sleep quality started as early as the 1970s [1]. Emerging data have shown that poor sleep quality is commonly seen after cardiac surgery, especially coronary artery bypass grafting (CABG) [2–9]. Poor sleep quality may impact patients’ recovery rate, quality of life, morbidity, and even mortality [2–5, 7]. Redeker et al. summarised the possible intrinsic and extrinsic factors that may influence sleep quality during hospitalisation and the recovery period. These include a number of things: age, gender, illness, primary sleep disorders, environment, medical treatment, etc. [7].

Infective endocarditis (IE), also called bacterial endocarditis, refers to cardiac infection caused by microbial invasion of the endocardium and involves the valves in most of cases [10, 11]. Studies have shown that approximately half of the IE patients require heart valve replacement surgery. Also, early surgical treatment in patients with surgical indications improves the prognosis [12–14].

To date, the literature related to the sleep quality of patients who have undergone heart valve replacement surgery is very limited. Our previous study showed that 71 out of 117 patients (60.7%) experienced lower sleep efficiency (< 85%) after heart valve replacement surgery [15]. Therefore, in the present study, we aimed to evaluate patients’ sleep quality after heart valve replacement surgery using both subjective and objective measures and also to investigate the possible risk factors for disturbed sleep quality during the post-hospitalisation recovery period.

## Methods

### Study population and design

Patients who underwent heart valve replacement surgery due to IE between October 2016 and September 2018 in our hospital were included in the current study approximately 2 weeks after the surgery. These patients were admitted to the Department of Cardiac Surgery for surgical treatment first and then were transmitted to the cardiac intensive care unit (CICU) for critical postoperative care. Once a patient’s condition stabilised and was approved upon review by cardiac surgeons, they were transmitted to our ward for postoperative antibiotic and recovery treatment. The inclusion criteria for this study were: (1) aged at least 18 years old; and (2) being able to express thoughts and feelings. Patients who were delirious, unconscious or in coma were strictly excluded. It was made clear that participation in this study was completely voluntary and did not affect any further medical treatment for any participants or non-participants. The current study was approved by the Ethics Committee of Guangdong Provincial People’s Hospital (No. GDREC2016222H (R2)) and was performed in accordance with the ‘Declaration of Helsinki’ and ethical standards. All patients provided a signed informed consent form.

In the current study, both subjective scale measures and objective actigraphy were employed to evaluate sleep quality. Subjective scale measures included the Pittsburgh sleep quality index (PSQI) and the Epworth sleeping scale (ESS), which were employed in the assessments for all included patients on their second day of being transmitted to our ward and also at 6 months after surgery. The objective method used in this study was actigraphy. All included patients were required to wear an Actigraph monitor on the wrist of their dominant side for a consecutive 2-week time period starting on the same day when they were assessed with the scale measures. During the same time period, the patients were guided to also keep a standard sleep diary for further reference.

### Subjective sleep measures

The PSQI is a self-reported questionnaire which evaluates sleep quality and disturbance over a 1-month time period retrospectively. Consisting of 19 individual items, the questionnaire generates 7 aspect scores, which are summed to one global score. The aspects include: subjective sleep quality, sleep latency, sleep duration, habitual sleep efficiency, sleep disturbance, use of medication, and daytime dysfunction. Each component is weighted on a 0 to 3 interval scale, which gives a total score from 0 to 21. A lower score indicates better sleep quality [16–18]. A result of PSQI ≥ 8 points indicates poor sleep quality and disturbance, while a PSQI < 8 points represents good sleep quality [19].

The ESS is a very short questionnaire designed to evaluate daytime sleepiness. The questionnaire consists of 8 questions, each of which illustrates a situation that the subject may encounter in daily life. The subjects are required to rate the possibility of falling asleep for each item from 0 to 3, with 0 being very unlikely. Similar to the PSQI, an overall score is obtained by summing the scores for all 8 questions. A total score ≥ 9 is considered indicative of daytime sleepiness according to the latest published guideline in China [20].

### Objective sleep measures

An Actigraph monitor is a wearable device which records a subject’s gross physical activity over a certain period of time. It was designed to collect information regarding the subject’s activity and stillness using a built-in accelerometer [21, 22]. The Actigraph GT3X + model (Actigraph, Pensacola, FL, USA) [23] was employed in this study to measure sleep latency, total duration of time in bed, total sleep time, nocturnal frequency of awakenings, duration of awakenings, and sleep efficiency. Sleep latency, or sleep onset latency, refers to the time required for the subject to transition from full wakefulness to sleep. Sleep efficiency was defined as the ratio of total sleep time to total time in bed as a percentage. A widely accepted normal range for sleep efficiency is above 85%.

After 2 weeks of monitoring, the Actigraphs were collected from patients together with their sleep diaries. ActiLife 6, which is a data analysis software package bundled with the Actigraph units, was used to download and analyse the data from the monitors. The function of ‘automatic interpretation and review’ was used for early-stage data processing. Based on the results from automatic interpretation, manual correction was conducted using the patients’ sleep diaries as references.

### Statistical analysis

Statistical analyses were performed using SPSS (version 22.0, SPSS Inc., Chicago, IL, USA). Descriptive statistics are presented as mean and standard deviation (SD) or median and interquartile range (25th and 75th percentile) where appropriate. Numerical data are presented as quantity and percentage (%). Subjective scale results and actigraphy data at different times were compared using Wilcoxon signed-rank test and McNemar test where appropriate. Univariable logistic regression analysis was performed to detect the possible risk factors for disturbed sleep quality at 6 months after surgery (*p* < 0.05). These factors were then analysed using a multivariable logistic regression model to obtain the final results. A *p* value of < 0.05 was considered statistically significant.

## Results

### Basic demographic and clinical characteristics

At the end of the recruitment, 80 patients were included in the current study. The average age of the study population was 42.8 ± 14.2 years. Among these patients, 67.5% (n = 54) were male, while 32.5% (n = 26) were female. The average body mass index (BMI) was 20.4 ± 3.1 kg/m^2^.

In the current cohort, 66 patients (82.5%) were diagnosed with cardiac comorbidities before surgery. These included 26 (32.5%) cases with rheumatic heart disease, 21 (26.3%) cases with congenital heart disease, 16 (20.0%) cases with degenerative valve disease, and 3 cases (3.75%) with other conditions. Nineteen cases (23.75%) were complicated by embolism identified via radiological assessments, including 12 cases of cerebral infarction, 3 cases of splenic embolism, 3 cases of limb arterial embolism, and 1 case of renal artery embolism.

All these patients had undergone surgical treatment, which included 73 cases of single valve surgery and 7 cases of multi valve surgery. Single valve surgery included mitral valvuloplasty (MVP) in 21 cases, mitral valve replacement (MVR) in 19 cases, aortic valve replacement (AVR) in 31 cases, the Bentall procedure in 1 case, and tricuspid valvuloplasty (TVP) in 1 case. Multi valve surgery included double valve replacement (DVP) in 4 cases, MVR complicated with TVP in 2 cases, and AVR complicated with TVP in 1 case.

All the patients completed the sleep scale assessments during hospitalisation and at 6 months after surgery. Also, all the patients provided actigraphy data for 2 weeks during hospitalisation. There was no major concern about wearing the Actigraph monitor among the patients. No adverse event or withdrawal was observed in this study. All demographic and clinical characteristics of the study population are presented in Table 1.

**Table 1:** Demographic and clinical characteristics of the study population (n = 80)

Gender (male)	54 (67.5%)
Age (years)	42.8 ± 14.2
BMI (kg/m^2^)	20.39 ± 3.13
Previous stay in CICU (days)	2.2 ± 1.5
Use of analgesic (cases)	21 (26.3%)
Use of hypnotics (cases)	26 (32.5%)
Preoperative WBC (*10^9^/L)	8.42 ± 3.81
Postoperative WBC (*10^9^/L)	19.39 ± 8.69
Preoperative Hb (g/L)	111.35 ± 23.96
Postoperative Hb (g/L)	101.68 ± 14.58
Preoperative Alb (g/L)	33.81 ± 5.88
Postoperative Alb (g/L)	32.42 ± 3.89
Preoperative CRP (mg/L)	23.33 ± 25.22
Postoperative CRP (mg/L)	40.65 ± 35.79
Postoperative PCT (ng/ml)	0.69 ± 1.33
Vegetation ≥ 10 mm	41 (51.3%)
Multi-valve involvement	7 (8.8%)
Complication of embolism	19 (23.75%)

Data are presented as mean and standard deviation (SD) or quantity and percentage (%)

Postoperative measurement of WBC, Hb, Alb, CRP, and PCT levels was performed 1 day after transmission to the ward for postoperative antibiotic and recovery treatment

*BMI* body mass index, *CICU* cardiac intensive care unit, *WBC* white blood cell, *Hb* haemoglobin, *Alb* albumin, *CRP* C-reactive protein, *PCT* procalcitonin

### Sleep quality assessment

The PSQI and ESS scale evaluation was conducted in all 80 patients approximately 2 weeks after surgery during hospitalisation and at 6 months after surgery. Analysis of the scale results showed that the median scores of the two scales were 6.5 and 5 points, respectively, at 6 months after surgery. These values were significantly lower than the median scores obtained during hospitalisation, which were both 7 points (both *p* < 0.001). The number of cases with a PSQI of ≥ 8 and an ESS ≥ 9 at 6 months after surgery were 35 (43.75%) and 11 (13.80%), respectively. There were no significances in the number of patients with a PSQI of ≥ 8 and an ESS of ≥ 9 during hospitalization or at 6 months after surgery (*p* = 1.000, *p* = 0.093). Additional results regarding the scale measures are presented in Table 2 and Additional file 1: Fig. S1.

**Table 2:** The PSQI and ESS scores at 2 weeks after surgery during hospitalisation and at 6 months after surgery (n = 80)

	During hospitalisation	Post-surgery	*p* value
PSQI (points)	7.00 (5.50, 11.00)	6.50 (4.00, 8.00)	< 0.001^*^
PSQI ≥ 8 (cases)	36 (45.00%)	35 (43.75%)	1.000
ESS (points)	7.00 (5.00, 8.50)	5 .00 (3.50, 7.00)	< 0.001^*^
ESS ≥ 9 (cases)	20 (25.00%)	11 (13.80%)	0.093

Data were analysed using Wilcoxon signed-rank test or McNemar test where appropriate. Data are presented as median and interquartile range (25th and 75th percentile) or quantity and percentage (%) where appropriate

*PSQI* the Pittsburgh sleep quality index (PSQI), *ESS* the Epworth sleepiness scale

^*^p value < 0.05

The actigraphy data showed that 43 out of 80 patients (53.75%) had a sleep efficiency greater than 85% during hospitalisation, and thus, for 37 patients (46.25%) the sleep efficiency was less than 85%. Generally, the median total time in bed was 492.7 min, the median total sleep time was 406.0 min, and the median sleep efficiency was 85.6%. The median sleep latency was 5.9 min, and the median frequency of nocturnal awakenings was 19.5 times. The median duration of awakenings was 73.5 min, with an average of 3.7 min each time.

Compared with the first week, significant differences were observed in sleep efficiency, sleep latency, total sleep time, frequency of awakenings and the number of cases with a sleep efficiency ≥ 85% in the second week. In week 2, sleep efficiency, the number of cases whose sleep efficiency was ≥ 85%, and total sleep time were significantly greater (*p* < 0.001; *p* = 0.046; *p* = 0.027) than those in week 1, while sleep latency and the frequency of awakenings were significantly lower (*p* < 0.001; *p* = 0.016). However, no significant differences were found for total time in bed (*p* = 0.100), duration of awakenings (*p* = 0.139), or average awakening duration (*p* = 0.881) between the first and second week. The actigraphy results are detailed in Table 3.

**Table 3:** Actigraphy results collected 2 weeks postoperatively during hospitalisation (n = 80)

Variables	Week 1	Week 2	Week 2–Week 1	*p* value
Sleep efficiency ≥ 85%	41.00 (51.25%)	49.00(61.25%)	–	0.046^*^
Sleep efficiency (%)	85.28 (79.75, 89.93)	86.76 (81.36, 90.38)	2.18 (− 0.75, 4.26)	< 0.001^*^
Sleep latency (min)	6.38 (5.46, 9.29)	5.04 (3.47, 7.99)	− 2.19 (− 3.50, 0.13)	< 0.001^*^
Total time in bed (min)	488.655 (460.93, 513.58)	499.79 (461.66, 525.25)	5.16 (− 14.36, 30.93)	0.100
Total sleep time (min)	398.91 (367.67, 439.57)	419.05 (380.49, 458.86)	5.98 (− 13.02, 44.58)	0.027^*^
Duration of awakenings (min)	71.49 (53.63, 96.62)	70.65 (50.76, 98.19)	− 4.84 (− 16.56, 11.95)	0.139
Average awakening duration (min)	3.58 (3.02, 4.60)	3.72 (2.88, 4.73)	0.06 (− 0.63, 0.64)	0.881
Frequency of awakenings	20.00 (16.22, 23.86)	19.34 (14.57, 23.29)	− 2.11 (− 4.23, 2.09)	0.016^*^

Data were analysed using Wilcoxon signed-rank test or McNemar test where appropriate. Data are presented as median and interquartile range (25th and 75th percentile) or quantity and percentage (%) where appropriate

^*^p value < 0.05

### Risk factors for poor sleep quality

When processing data related to potential risk factors for poor sleep quality in the posthospitalisation period, univariable logistic regression analysis showed that age, PSQI score during hospitalisation, sleep efficiency, total sleep time, duration of awakenings, and frequency of awakenings may be relevant (all *p* < 0.05; Table 4). With these variables taken into consideration, multivariable logistic regression analysis revealed that only four were statistically significant, including age (odds ratio [OR] = 1.191, 95% confidence interval [CI] 1.082–1.311), early postoperative PSQI score (OR = 1.646, 95%CI 1.226–2.210), early postoperative sleep efficiency (OR = 0.607 95%CI 0.433–0.852), and average duration of awakenings (OR = 0.960, 95%CI 0.922–1.000). The complete results are presented in Table 5.

**Table 4:** Univariable logistic regression analysis exploring potential risk factors for disturbed sleep quality at 6 months after surgery (n = 80)

Variable	B	*p* value	OR	95% CI
Gender	− 0.144	0.764	0.866	0.338–2.218
Age	0.079	< 0.001^#^	1.082	1.039–1.127
BMI	0.075	0.306	1.078	0.933–1.246
Previous stay in ICU (days)	0.092	0.549	1.096	0.811–1.482
Use of analgesic (cases)	− 1.005	0.055	0.366	0.131–1.022
Use of hypnotics (cases)	− 0.144	0.764	0.866	0.338–2.218
Preoperative WBC (*10^9^/L)	0.066	0.307	1.068	0.941–1.213
Postoperative WBC (*10^9^/L)	− 0.002	0.939	0.998	0.948–1.050
Preoperative Hb (g/L)	− 0.015	0.129	0.985	0.966–1.004
Postoperative Hb (g/L)	− 0.028	0.088	0.972	0.941–1.004
Preoperative Alb (g/L)	− 0.032	0.414	0.969	0.898–1.045
Postoperative Alb (g/L)	− 0.077	0.198	0.926	0.823–1.041
Preoperative CRP (mg/L)	0.003	0.776	1.003	0.985–1.020
Postoperative CRP (mg/L)	− 0.001	0.867	0.999	0.987–1.011
Postoperative PCT (ng/ml)	− 0.498	0.091	0.608	0.341–1.083
Vegetation ≥ 10 mm	0.191	0.673	1.210	0.500–2.931
Multi-valve involvement	− 0.0591	0.460	0.554	0.115–2.653
Complication of embolism	0.088	0.869	1.092	0.385–3.094
PSQI during hospitalisation (points)	0.166	0.008^#^	1.181	1.045–1.334
ESS during hospitalisation (points)	0.021	0.741	1.021	0.902–1.156
ESS ≥ 9 during hospitalization	− 0.068	0.896	0.935	0.338–2.587
Sleep efficiency (%)	− 0.200	< 0.001^#^	0.819	0.740–0.905
Sleep latency (min)	0.073	0.345	1.076	0.924–1.252
Total time in bed (min)	− 0.012	0.069	0.988	0.976–1.001
Total sleep time (min)	− 0.015	0.001^#^	0.985	0.976–0.995
Duration of awakenings (min)	0.023	0.002^#^	1.025	1.009–1.041
Frequency of awakenings	0.110	0.015^#^	1.116	1.022–1.219
Average duration of awakenings (min)	0.263	0.073	1.300	0.976–1.732

Data were analysed in univariable logistic regression

*OR* odds ratio, *95% CI* 95% confidence interval, *BMI* body mass index, *ICU* intensive care unit, *WBC* white blood cell, *Hb* haemoglobin, *Alb* albumin, *CRP* C-reactive protein, *PCT* procalcitonin

^#^*p* value < 0.05

**Table 5:** Multivariable logistic regression analysis to identify potential risk factors for disturbed sleep quality at 6 months after surgery (n = 80)

Variable	B	*p* value	OR	95% CI
Age	0.175	0.000^*^	1.191	1.082–1.311
PSQI during hospitalization (points)	0.498	0.001^*^	1.646	1.226–2.210
Sleep efficiency (%)	− 0.499	0.004^*^	0.607	0.433–0.852
Total sleep time (min)	0.006	0.634	1.006	0.982–1.030
Duration of awakenings (min)	− 0.041	0.048^*^	0.960	0.922–1.000
Frequency of awakenings	0.108	0.238	1.114	0.931–1.332

Data were analysed in multivariable logistic regression

*OR* odds ratio, *95% CI* 95% confidence interval

^*^p value < 0.05

## Discussion

To our knowledge, the current study is the first one designed to evaluate patients’ sleep quality after undergoing heart valve replacement surgery due to IE using both subjective and objective measures. The literature relevant to the topic of patients’ sleep quality after cardiac surgery is rather limited, and most studies to date have focused on CABG cases only [2, 4, 5, 8, 18, 24–27]. These studies investigated sleep quality in patients who underwent CABG surgery, either over a short-term or a longer period of up to 6 months. Use of a scale measure/questionnaire on site or via telephone interview was commonly seen in these studies. A few of them did however use both subjective and objective measures for evaluation [2, 6, 9, 18, 26, 27]. Sleep efficiency was one of the common interests shared in these studies, which was usually based on actigraphy measurement. Redeker et al. [6] reported that the mean sleep efficiency was 71% at 4 weeks and 74% at 8 weeks postoperatively. In the study by Yilmaz and Iskesen [9], it was revealed that the mean sleep efficiency was 66.55 ± 11.00% for male and 67.80 ± 13.72% for female at postoperative first week, and 94.01 ± 0.94% for males and 94.10 ± 1.21% for females at postoperative second month. Our previous study [15] showed that, of 117 patients with an average sleep efficiency of 80.81 ± 8.52%, 71 (60.7%) had sleep efficiency below 85%. The findings from the current study suggest that the overall postoperative sleep efficiency about 2 weeks after surgery during hospitalisation was 85.6%, which is consistent with the results obtained in the earlier studies.

Previous studies also attempted to include multiple evaluation time-points to monitor changes in sleep patterns after cardiac surgery and to discover when a significant improvement could be observed. Schaefer et al. [5] conducted telephone interviews with patients at 1 week, 1 month, 3 months and 6 months after surgery. According to the patients’ feedback, sleep disturbance occurred less often as more time passed. Redeker et al. [6] observed sleep disturbance rates of 64% at 4 weeks and 47% at 6 weeks after cardiac surgery. In another study by Redeker et al. sleep disturbance was observed to be higher at 1 week than at 4 weeks and at 8 weeks after surgery [26]. An even earlier publication by the same research team also supported the idea that more consolidated night sleep was seen in the late posthospitalisation period rather than early stage as 1 week after surgery [27]. In the present study, for short-term outcomes, sleep efficiency and a few parameters measured by actigraphy were significantly improved in week 2 when compared to week 1. For longer term outcomes, both the PSQI and ESS results were also significantly lower at 6 months after surgery than during hospitalisation. It is worth noting that disturbed sleep mainly manifested as frequent micro awakenings at early postoperative period. These results together with those of previous studies indicate that sleep quality is disturbed during the early postoperative period, but a trend of gradual recovery in sleep quality occurs over time after surgery.

Research findings have shown that disturbed sleep quality is a process affected by multiple, correlating factors [7]. It also has been well accepted that surgical trauma, anaesthesia, admission, etc., which all patients who undergo any surgery will experience, have a severe impact on patterns of sleep quality [28, 29]. Our results from the current study showed that age, early postoperative PSQI score, sleep efficiency, and average duration of awakenings are risk factors for poor sleep quality at 6 months after cardiac surgery for IE. The latter three variables are not singular but rather are affected by many other factors. A low early postoperative PSQI score may be related to all of the surgical elements mentioned above, and possibly a change in environment or postoperative complications, if they occur. Similar explanations can be applied to early postoperative sleep efficiency and average duration of awakenings as well. Therefore, a more precise way of analysing how severely these potential risk factors contribute to disturbed sleep quality will be a question for future studies.

Apart from using both subjective and objective measures for sleep quality, another advantage of the present study is that we chose the study time to be about 2 weeks after the surgery during hospitalisation. In this case, the effects of anaesthesia were almost completely eliminated. Also, because the patients had stabilised before being transmitted to our ward, any impact from early postoperative reactions, such as severe pain or inability to move, was also minimised.

This study also has limitations. We were unable to collect information for all patients’ preoperative sleep quality and psychological status. This flaw in the study design was due to the nature of our ward, which is allocated to stabilised postoperative patients only. Therefore, we are unable to draw the conclusion that sleep efficiency was worse postoperatively versus preoperatively, even though this was suggested by other studies [2, 27]. Secondly, this study was performed in patients after valve replacement for IE, which might reduce the heterogeneity and also limits the generalisability of the findings. Another limitation, which was discussed before, is the selection of potential risk factors for logistic regression analysis. Future studies are needed to expand our knowledge of how much these different risk factors contribute to the onset of disturbed sleep quality.

## Conclusions

In the current study, we employed both subjective and objective measures to evaluate sleep quality in patients who underwent heart valve replacement surgery. Disturbed sleep quality was witnessed in early postoperative period and up to 6 months after surgery. However, an improvement in sleep quality was also observed over the recovery period. Age and a few early postoperative sleep quality variables, including PSQI score, sleep efficiency and average duration of awakenings, may indicate poor sleep quality in the later recovery period. Interventions to improve patients’ sleep during hospitalisation and posthospitalisation are in urgent need.

## Supplementary Information

**Additional file 1: Fig. S1.** The PSQI and ESS scores at 2 weeks after surgery during hospitalisation and at 6 months after surgery.

## Data Availability

The datasets generated and analyzed during the current study are available from the corresponding author upon reasonable request.

## Abbreviations

CABG: Coronary artery bypass grafting
IE: Infective endocarditis
CICU: Cardiac intensive care unit
PSQI: The Pittsburgh sleep quality index
ESS: The Epworth sleeping scale
SD: Standard deviation
PCT: Procalcitonin
WBC: White blood cell
Hb: Haemoglobin
Alb: Albumin
CRP: C-reactive protein
OR: Odds ratio
