# Supplementary material for: Sleep patterns and potential risk factors for disturbed sleep quality in patients after surgery for infective endocarditis
Source: J Cardiothorac Surg. 2022 May 17;17:121. doi: 10.1186/s13019-022-01828-4 (PMC9116038; doi:10.1186/s13019-022-01828-4)
Supplement: Supplementary file 1 — Additional file 1: Fig. S1. The PSQI and ESS scores at 2 weeks after surgery during hospitalisation and at 6 months after surgery. [file 13019_2022_1828_MOESM1_ESM.docx]

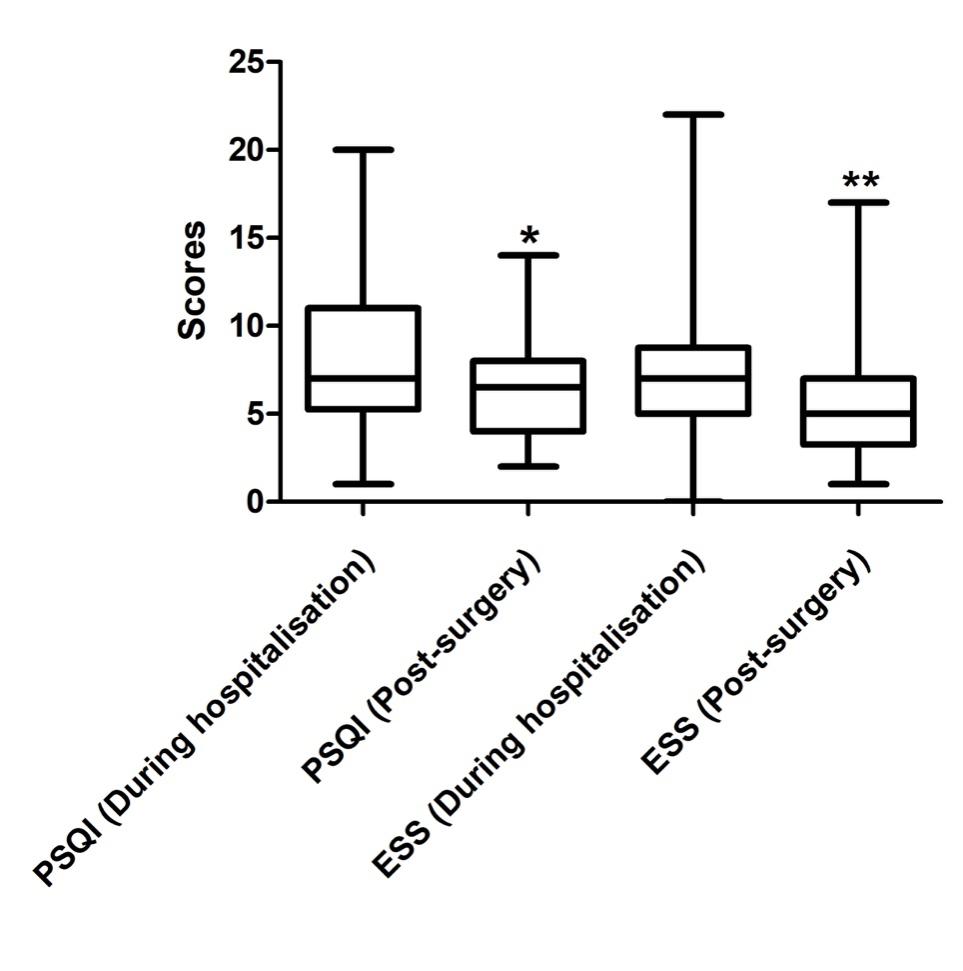


**Additional file 1: Fig. S1.** The PSQI and ESS scores at 2 weeks after surgery during hospitalisation and at 6 months after surgery
